# Community-Driven Priorities in Smartphone Application Development: Leveraging Social Networks to Self-Manage Type 2 Diabetes in a Low-Income African American Neighborhood

**DOI:** 10.3390/ijerph16152715

**Published:** 2019-07-30

**Authors:** Pamela J. Surkan, Kathryne S. Mezzanotte, Laura M. Sena, Larry W. Chang, Joel Gittelsohn, Ylva Trolle Lagerros, Charlene C. Quinn, Wayne W. Zachary

**Affiliations:** 1 Department of International Health, Johns Hopkins Bloomberg School of Public Health, Johns Hopkins University, Baltimore, MD 21205, USA; 2 Department of Medicine, Johns Hopkins University School of Medicine, Johns Hopkins University, Baltimore, MD 21205, USA; 3 Department of Medicine, Karolinska Institute, Clinical Epidemiology Unit, Stockholm, and Obesity Center, Academic Specialist Center, Stockholm Health Services, 113 64 Stockholm, Sweden; 4 Department of Epidemiology and Public Health, University of Maryland School of Medicine, Baltimore, MD 21201, USA; 5 Starship Health Technologies, LLC, 610 Sentry Parkway, Blue Bell, PA 19422, USA

**Keywords:** diabetes, mHealth, type 2 diabetes mellitus, social network, mobile application

## Abstract

Social networks have the potential to enhance Type 2 Diabetes Mellitus (T2DM) self-management. We used qualitative methods to study if and how mobile application (app) functions that mobilize social resources to improve T2DM management would be desired in a low-income African American community. Data were collected through community discussions and in-depth interviews with 78 participants in 2016–2018. Participants included individuals with self-reported pre-diabetes, T2DM, close family members or friends of a T2DM patient, and healthcare providers. Open-ended questions solicited information about challenges with T2DM management and gathered ideas on features of a mobile app that could address them. Data were transcribed and thematically coded by two coders using Atlas-ti. Regarding types of app functions, main themes included: (1) the importance of having support in diabetes self-care; (2) using informal networks to help to each other; and (3) monitoring one another through an app. Suggested app features included reminders for and transportation to medical visits, sharing information and exercise companionship, and providing opportunities for monitoring by friends/family members, especially in case of emergencies. Participants viewed an app as a potential vehicle for reinforcing accomplishments in T2DM self-management. Future research should implement and test an app with these features in this or similar communities.

## 1. Introduction

Diabetes mellitus is a chronic metabolic disease that requires ongoing glycemic control and medical care [1]. Affecting 30.3 million, or 9.4%, of the U.S. population, diabetes was the seventh leading cause of death in 2016 and contributed to 80,058 U.S. deaths [2]; an additional 33.9% or 84.1 million American adults are pre-diabetic [2]. Type 2 Diabetes Mellitus (T2DM) accounts for 90–95% of all diabetic cases and its prevalence has been rising among low-income minority communities [3,4,5]. Poor glycemic control due to such socio-environmental influences means that African Americans diagnosed with T2DM are more likely to develop severe complications [6].

The American Diabetes Association (ADA) recognizes the need for greater attention to both the psychosocial factors and the social context that influence diabetes-related outcomes [7]. Risk for T2DM and a patient’s subsequent ability to self-manage T2DM are affected by features of the social environment [8], e.g., neighborhood safety, healthy food availability, and transportation [9]. Despite their influence on disease management [10,11], social networks are a relatively neglected pathway to self-care for people with T2DM. Social support is a predictor of self-care habits, including consistent exercise and medication adherence that reinforce diabetes management [12]. Family and friends can reinforce healthy behaviors through supportive messaging, observational learning, offering new skills and knowledge, and helping to create healthier household food environments [13].

Social support has been conceptualized as reflecting four domains: emotional, instrumental, appraisal, and informational support [14]. Emotional support represents love and caring, while instrumental support refers to aid or assistance in tangible activities [15]. Appraisal support can be viewed as a type of informational support, but involving self-appraisals, while the concept of informational support refers to the receipt of information/advice [15].

Social networking technology is developing at an impressive pace [16,17]. Recent advances in mobile health (mHealth) have created new opportunities to improve self-management of T2DM through tools to facilitate healthy eating, exercise, and access to healthcare. One such innovative model involves integrated systems that connect patients via mobile smartphone to their support networks [18]. However, to date, little research has focused on how smartphone applications (apps) can harness technology to support T2DM patients in growing a support network.

A 2018 review of 173 T2DM apps available for Apple iOs and Google Android platforms found that the majority were designed to support healthy eating, monitoring, medication reminders, and being active [19]. A 2017 review of 35 T2DM apps available in the Apple App Store and Google Play found that most were focused on reporting and setting reminders instead of providing personalized education or therapeutic support [20]. Social aspects and social media components remain underused in T2DM apps [21]. A search of existing commercial apps identified only five (Health2Sync, Healthseeker, Sugar Sense, One Drop, and eCoFit) as having any social component, and those only in limited ways, not involving first-order network social support.

Given the potential of social networks to improve diabetes care, our study was conducted with participants from a low-income African American neighborhood to understand their perceptions of how a mobile app could help people mobilize social resources to improve diabetes self-management. The aim of this study was to use a community perspective to explore how types of social support and social networks could be used to develop an app to help with T2DM self-management.

## 2. Materials and Methods

### 2.1. Participants/Setting

Inclusion criteria for study participants included people with T2DM and their friends and family members who were: English-speaking, age >18, and primarily residing in the designated low-income study communities. We studied the Southwest Baltimore communities of Sandtown/Winchester/Harlem Park because of our prior research in the neighborhood indicating that individuals share information about diabetes self-management through social networks [13], and that mobile devices are widely used [22]. Furthermore, diabetes is the sixth leading cause of death in these communities, accounting for 4.2% of all deaths in 2017 [23]. The overall median income for Baltimore was $41,819 in June 2017, while for the study communities it was $24,374 [23,24]. 96.7% of these neighborhoods’ residents were African American in 2017 [23].

Information from patients and family members was collected primarily through community forums, i.e., interactive sessions that required a high degree of engagement, and through some in-depth interviews. Twenty health care providers participated in either in-depth interviews or a focus group discussion. After app design concepts were created from community forum and provider data, additional patients and family/friends participated in pre-usability forums to discuss design concepts. Recruitment was spaced over the course of two years for two main reasons: (1) difficulties in recruitment and (2) simultaneous development of an app prototype that was iteratively based on feedback. Many community members had multiple part-time jobs, and little access to affordable transportation, which made it difficult to find times when people were available and to find locations that were easily accessible for people who were interested in participating. Simultaneous technological development of the app was also conducted in parallel to, and in response to, the collection and analysis of the data. Therefore, data collection was paced to coordinate with the development of app features.

Consistent with the high incidence of T2DM in the community, some non-provider participants with pre-diabetes or T2DM were also friends/family members of other participants with pre-diabetes or T2DM. For categorization, such participants are reported here as adults with pre-diabetes or T2DM. All but one non-provider participant self-identified as African American. Most (*n* = 59) participated in one of six discussion group forums (averaging in size *n* = 9 participants), but a few non-provider individuals participated in one-on-one interviews (*n* = 4). Four individuals from early forums also participated in later pre-usability forums.

Data from providers were collected through nine individual in-depth interviews (*n* = 9), one focus group (*n* = 9), and one paired interview (*n* = 2). Providers included diabetes educators, pharmacists, pharmacy student, nurses, and a physician’s assistant, as well as physicians (primarily from health care facilities in the study area).

The study took place from February 2016 to May 2018, and included 7 forums and 15 in-depth interviews. Participant recruitment was done in collaboration with community organizations (e.g., residential buildings, a neighborhood supermarket), primarily through distributing flyers and describing the project to community members attending meetings, community events, and shopping at a local supermarket. Forums generally lasted 3 h, while interviews lasted approximately 1 h. Forum participants were given a $50 or $40 gift card, depending on the length of the forum, and $30 for participation in interviews.

Forums consisted of a series of sequential, interactive group discussions around experiences and barriers/problems to T2DM self-management, from either a first-person perspective or that of a close family member or friend, with the stated goal of creating a smartphone app that could help with T2DM self-management. A moderator (with qualitative research training at the graduate level) used a semi-structured discussion guide and was accompanied by a research team (representing the fields of public health, anthropology, and computer interface design) that took notes. The open-ended discussion guides asked about common problems with diabetes self-management and solicited ideas regarding features of the mobile app that could be used to help with such challenges.

The principle goal of the first set of forums was to gather ideas about features of the app that would be useful to users, while later ‘pre-usability’ forums aimed to achieve this goal while also reviewing and refining design concepts developed from earlier forum data. We used active moderation as well as structured record keeping based on audio recordings, verbatim transcription of all recordings, and digital photographs of artifacts (e.g., group-generated lists of desired functions, constraints on the design, drawings, sketches of how a diabetes app should be organized). Forums were supplemented by in-depth interviews with four community residents with T2DM. Interview topics were similar, focusing on neighborhood barriers and facilitators to T2DM self-management. Interviewers also solicited participants’ ideas about which features of an app could be useful for them.

### 2.2. Data Analysis

Data analysis was based on thematic content analysis [25]. We primarily used an inductive approach to explore: (1) major areas and sub-areas of problems in diabetes self-management; and (2) ideas/solutions that could be implemented on a mobile app.

Data transcription was concurrent with data collection, and themes were reviewed and discussed regularly by the research team, so that later forums and interviews could build on earlier findings [26]. Coding was undertaken by two study team researchers, with initial coding done with Atlas-ti computer software (Version 7, Scientific Software Development GmbH, Berlin, Germany), followed sequentially by further code refinement by a second team member. Additional themes evolved throughout the analytic process, as more patterns were identified. Multiple coders allowed for the discussion of differences to reach consensus. Memos were used throughout the analysis to document and share intermediate results, and the constant comparative method was used to facilitate theme development [27,28].

The Johns Hopkins Bloomberg School of Public Health (FWA 00000287; IRB X 00000758)) determined the study protocol to be Exempt, Category 2 (45 CFR 46.101(b)). This designation was made based on the fact that participants were not identified in recorded data and that disclosure of human subject responses would not place the participants at risk. All participants provided informed consent prior to participation.

## 3. Results

We conducted qualitative research with 78 participants to inform the development a mobile phone app for people with T2DM. Fifty-eight were self-identified as pre-diabetic, as having T2DM, or self-identified as being a close family member or friend of a T2DM patient who helps with diabetes care.

Participants included adults with self-reported pre-diabetes or T2DM (*n* = 31), close family/friends of people with T2DM (*n* = 27), and healthcare providers in the study community (*n* = 20) (see Table 1).

An overarching theme was the importance of social support for diabetes self-management in this community. Themes also emerged around ways that an app could be used; these focused mainly on motivating healthy behaviors, providing support to each other, and monitoring each other’s progress (see Table 2 for all themes and subthemes).

### 3.1. Importance of Having Support in Diabetes Self-Care

A major theme was the importance of social support for T2DM management. Many participants highlighted how having social support helped them maintain healthy behaviors around both exercise and nutrition.

“Everyone can piggyback off one another and develop not just healthy eating, but exercise together…Knowing you have a network is huge, because then you are not alone.”

*(Forum 4)*

Most participants viewed an app as a potential way to foster support to help them take part in diabetes management activities like exercise.

“Now, not all of us [are] living in the same places so we need this app. I believe [it] is real good…we can exchange like if Stanley is online I can send him a text or something if he’s online like we can meet up somewhere—let’s get up with Chrystal—let’s get up with Miss Jackie and lets go for a walk.”

*(Forum 1)*

Other participants stated how exercising with friends can be motivating and enjoyable.

“It fun, you don’t even realize you’re working out, doing it for your health...we need that support to help motivate us, to get some of the stress, to exercise, and to be in better health.”

*(Forum 1)*

Some mentioned the positive impact of social support on food choices.

“When you make the commitment to yourself—get two or three other people and you have a network. You all have each other’s support... You gonna need some support. So, if you set up a support, you stand a better chance of not buying the food we don’t need to buy.”

*(Forum 3)*

Several providers reinforced this idea that an app could motivate self-care of T2DM by enhancing social support through companionship. One provider explained:

“The other thing that anecdotally that patients tell me is that ‘I’m not motivated to go by myself,’ but if they have a partner or buddy or somebody [things would be different.]”

*(Provider Interview)*

### 3.2. Using Informal Networks to Help Each Other

A number of participants provided ideas for how a support network could facilitate reminders and transportation for going to healthcare visits. As one provider suggested:

“Like any chronic condition, if you have people around you who can help you, remind you to ‘do this,’ ‘this thing’s bad for you,’ [and provide] some family support. Some people have no one to take them to see the doctor when they need to see the doctor, and that could be a problem.”

*(Provider Interview)*

Motivation through contact with friends and family was echoed throughout the data. As two participants illustrated:

P1: “I find, myself, when I do things with someone [then] I really stick to it, but if it’s just me myself I just lay on the couch.”

P2: “That’s why I was saying [about] the app—this app would be good because everyone who’s in the group and others who come along—we could connect through the app.”

*(Forum 1)*

### 3.3. Monitoring Each Other Through the App—Checking Blood Sugar

Participants described how an app could be used to check on others. As one woman stated:

“That’s how we know who’s cheating, and who’s not checking their sugars and things like that, in keeping a record, you can see when I did my last finger stick, so I know what it was. So I know if that I know at 12 o’clock your last finger stick was 80 I’m gonna send you a text like ‘Look girl, I know your last finger stick was 80, how you doing? Is everything okay?…This app, it can go so many places.’”

Another participant described challenges with finger sticks and suggested how a webcam might be used to connect people with T2DM to each other. For example, the app could be used to virtually share experiences:

“So maybe you have this device where I can come on my phone and I go, ‘You ready to do your finger stick? Okay, let’s do them together.’ She has her phone. I have mine. We got the camera. 1, 2, 3...stick. Now we got our numbers. I know she did it. She knows I did it because we seen each other doing it.”

*(Pre-Usability Forum)*

### 3.4. Monitoring Each Other Through the App—Taking Medication

Much discussion in the forums and in-depth interviews focused around use of the app as a way to monitor each other’s T2DM self-care in terms of medication. One idea was that it could be used to coordinate reminders among network members.

It would be nice if it [my brother’s phone] were shared with my phone so that even if he gets a reminder on his smartphone it also sends a reminder to my phone, so if he doesn’t do what he needs to do then I can call him and check on him that ‘Hey, I just got a reminder that you’re supposed to take your medicine, did you do that?’

*(Forum 4)*

Some participants suggested that they could use the app to get support for equipment needed to aid in diabetes care.

“Let’s say like if I ran out of test strips and the kind that I use I can’t buy over the counter, although you can, but something of that nature. I’m in an emergency, I am having an emergency. What if there’s some type of app or within your app that I could reach out in my community and say, ‘Hey I’m out of testing strips, I’m out of insulin,’ or whatever. Is there anyone that I can go to for like a borrow? Not personally but like are there resources—like are there pharmacies that will do something?”

*(Pre-Usabilty Forum)*

Others expanded on potential use of a buddy system to help T2DM patients and their contacts keep track of medications.

“Seeing as is you take your medicine every day—how you gonna keep track? With the app, instead of keeping track on a notebook or something, you can keep track on the app so as though your family members, or whoever you have as an emergency contact, you know can see you is taking your medicine and as I said you can get a reminder sent to your smartphone, like ‘oh they haven’t responded to the reminder but you see online that they have tracked in that they took their medicine and did everything they’re supposed to do, they just didn’t respond to the reminder.’”

*(Forum 4)*

### 3.5. Sharing Information about T2DM with Others Through the App

Participants also suggested sharing information through the app. As one woman suggested *“Postings of foods that are low in fat. I screenshot that—because I think that’s important. It’s more of, like, a sharing.”* Others suggested taking advantage of social networks through the app to *“send recipes back and forth,”* along with posting information about activities, community groups and resources, to increase physical activity. One woman described:

“With the app would it be possible so like you could drop like a pin if you went to something helpful. So instead of just having the calendar and the addresses in—it might make it more easier.”

*(Pre-Usability Forum)*

Others suggested posts could include diabetes-friendly dishes that they have prepared.

“The group can discuss, ‘Well this is what I cooked for dinner, what I put together from this,’ and that would be a good idea.”

*(Provider Interview)*

Healthcare providers however cautioned about the possibility for misinformation:

“I think that’s great both for self-efficacy and I think that’s a huge way to increase motivation, but I think I would just be cautious about that with the potential for misinformation. I don’t know how that’s monitored. I think it’s okay to share recipes, but from what I understand, here is such a knowledge deficit of what is truly healthy.”

*(Provider Interview)*

However, participants also discussed ways to circumvent misinformation.

“Maybe you could have a toll free number on the bottom” in order to “call someone who you know who actually has experience.”

*(Pre-Usability Forum)*

### 3.6. Using the App to Help Each Other in Case of Emergency

Many themes from discussions in the forums and interviews revealed how support could be mobilized in an emergency. Another popular potential use of the app included developing a system of automatic alerts to be sent to emergency contacts.

“You can keep in the application…someone who is very close to you’s contact number like email or phone number and we can send a message to them…and in emergencies we can send a message.”

*(Pre-Usability Forum)*

In the context of worrying about someone to help in a crisis, one woman suggested:

In developing the app, like you’re talking about, each and every one of us in this community in this forum and we could exchange information so that we know ‘Okay well I haven’t heard from Babette she usually online at 7 am she gets off at 11 am to watch The Price is Right, she checks back in at 2, so if I haven’t heard from Babette at 2 o’clock its now 5 o’clock, so that mean I’m going to hit everybody in the forum to ask them, have you guys spoken from Babette? ‘No I haven’t talked to her, she hasn’t been online,’ then my next step is to get to a phone, call her son, call her fiancé, and say ‘Look—is Babette okay?’ ‘Yeah she’s fine, she just didn’t feel good after lunch.’ So I can get back online to the community or forum and say I talked to Babette’s family. She okay. She just wasn’t feeling well after lunch.”

*(Forum 1)*

Other variants on the idea of having an alert on the phone to signal an emergency were proposed.

“Is there something that could be on your phone that you haven’t checked in or checked your messages so anything within 3–4 h that would set an alert to you or a sister?”

*(Forum 4)*

There was general agreement that such an alert could go to a caregiver like a sister, grandmother, or another supportive person who could get to the person with T2DM quickly if necessary. Expanding on this point, another woman suggested *“You wouldn’t have to say anything. It [could be] just a button to know if someone could come to you, just to let you know he’s woke and aware.” (Forum 4)*

### 3.7. Reinforcing Each Other’s Accomplishments through the App

Another theme included using the app as a way to showcase one’s accomplishments and motivate others. One man explained:

“Like we was saying with the calendar, if your friends or family can see you’ve been doing good, they can give you encouragement on the calendar. If they see you’ve been taking your medicine on time lately—congratulations!”

*(Forum 4)*

Other providers agreed, adding:

“It becomes motivation, when someone tells you my A1c used to be 9.6 and I changed to do something, and do you believe it now? I’m on the 7, or ‘I used to take two or three medications but I started 30 min walking—just a simple 30 min walk in the evening and now I’m taking one or two.′ That can be a sort of motivation that they might listen to.”

*(Provider Interview)*

Others suggested sharing information about diabetes management, such as glucose values, with other app users, as a way to encourage others.

“Like I said, I was 210. From 210 to 153…and you can put that down—that that’s what you accomplished, so everyone in your friends, your circle, know it’s an accomplishment.”

*(Forum 1)*

## 4. Discussion

To our knowledge, this is the first study to investigate which features of an app for T2DM self-management are prioritized within a low-income African American community with a high disease burden. We found that people with T2DM and their friends and family perceived that a social networking app would be valuable for T2DM management. They identified various functions of a potential mHealth app that could facilitate diabetes self-care, and those functions tapped into multiple different forms of social support. Themes were cross-cutting, and no differences were found between persons with pre-diabetes/T2DM and health providers.

Instrumental support emerged as the most common type of support that such an app could facilitate. Examples included finding someone to exercise with, to get reminders from, to help with transportation, to aid in finding equipment for diabetes care, or to check on one another. It is possible that receipt of instrumental support may work in tandem with emotional support (e.g., discussions about T2DM management might be facilitated while getting a ride from someone to a doctor’s appointment). That is, dual benefits may result from interacting with others about T2DM. Although less frequent, the app was discussed as a potential means for receiving informational support e.g., through sharing information about neighborhood resources or diabetes-friendly recipes. Ways that the app could enhance appraisal support were uncommon, though in a few forums, it was discussed how a T2DM app could enable feedback from others to enhance self-management.

Data suggested that tapping into social networks was perceived as critical for diabetes self-care. This idea reinforces research that suggests ways in which a patient’s social networks can support self-management, including through the sharing of illness narratives [29], direct influence on behaviors [30], help with diet and medication reminders, providing transportation [31], and increasing exercise and foot examinations [32]. Notably, although mHealth interventions have been proliferating, a recent systematic review found that app functionality is often limited to data upload, education, and text message notifications [33].

Some participant suggestions could be considered problematic with regard to translating them into a smartphone app design. One example is the idea of creating an exchange mechanism to share diabetes supplies (e.g., insulin or testing strips). Sharing diabetes-related resources is in conflict with published guidelines of both the Centers for Disease Control and the Food and Drug Administration [34,35], which prohibit sharing of blood glucose monitoring supplies/devices in order to prevent the transmission of infection and blood-borne pathogens [34,36]. Furthermore, types of medications and doses are personal and prescribed by a physician (e.g., using the same dose, but another type of insulin could be dangerous). Another suggestion raising ethical issues was the request for an alert mechanism on the app to notify family/friends of an emergency. Ethical and legal barriers to developing such app functions exist, if the emergency function failed to operate as intended. Therefore, such features should not be recommended for implementation.

One limitation was the inability to distinguish between people with pre-diabetes/T2DM and their friends and family members in our transcripts. A major study strength was the use of information solicited directly from people with pre-diabetes/T2DM and their first order networks. Complementing information from these participants with that of providers allowed us to triangulate findings and look for consistencies and inconsistencies across these groups. Within a high risk demographic area, capturing a sub-population disproportionately affected by T2DM and with limited socio-economic resources, we were able to gather specific information about useful features of a social-network-focused T2DM app, helping to bridge a gap between research and practice [37]. While our findings are relevant to this population, these findings may not be transferable to other socio-economic/cultural groups or rural settings. Although more men than women are affected by T2DM, our participants were predominantly women. It is unknown if our results would apply to men.

## 5. Conclusions

In summary, we found that a social network app for T2DM was considered a useful tool for a population of low-income African Americans, a high risk group for disparities in diabetes outcomes and care. Given that few diabetes apps have been evaluated in the peer review literature [21,38,39,40], future studies are needed to implement these ideas and to test their usage.

## Figures and Tables

**Table 1:** Participant characteristics of persons with prediabetes, Type-2 diabetes (T2DM), and friend and family member participant characteristics and types of data collection, *n* = 78.

Data Collection Type (Forum or Interview)	Participants, Number	Men, Number	African American	Prediabetes/Diabetes Type 2	Data Collection Date
T2DM and Friend/Family(F/F)					
Forum 1	9	2	8	9	17 February 2016
Forum 2	11	3	11	6	22 February 2016
Forum 3	18	9	18	0	14 April 2016
Forum 4	7	2	7	3 ^1^	26 July 2016
Pre-usability forum 1	6	2	6	4	2 December 2016
Pre-usability forum 2	7	3	7	6	2 June 2017
Persons who participated in 2 forums	4	1	3	3	
In-depth interviews					
Four in total	4	1	4	3	9 March 2017 10 March 2017; 1 April 2017; 13 April 2017
Total: T2DM and F/F	58	21	57	28	February 2016–July 2017
Providers					
Forum 5	9	5	5	N/A ^2^	18 May 2016 to 4 May 2018
Interviews	11	5	5	N/A	
Total: Providers	20	10	10	N/A	(see above)
Grant Total:	78	31	67	N/A	February 2016–May 2018

^1^ One participant did not provide information on race, gender, or T2DM status, but did present as African American and Female and is recorded as such. As this person’s T2DM status was unknown, no status was estimated, and the value for this person was recorded in the table as ‘not’ having T2DM. ^2^ Not Applicable (N/A) in this table means that providers were not asked this information and therefore it is unknown.

**Table 2:** Summary of themes and subthemes that emerged from the data.

THEME	SUBTHEME	DETAILS or EXAMPLE
Importance of having support in diabetes self-care	Exercise	*Motivating/enjoyable/safer to exercise with others*
	Nutrition	*Help in selecting appropriate foods*
Use of informal networks to help each other	Reminders	*For medications; For medical visits*
	Transportation	*Companionship to doctor’s appointments*
	Exercise	*Finding other persons to exercise with*
Monitoring each other through the app	Check on others’ self-monitoring	*Blood sugar checking; Sharing of diabetes care experiences*
	Check on others’ medications	*Coordinate with network members to keep track of medications, take them on time, get diabetes equipment needed*
Sharing information about T2DM	Sharing successful strategies	*Ideas for diabetes friendly foods/recipes that other participants have used*
	Sharing personal stories	*Strategies for dealing with difficult situations (e.g., food-intensive holidays) that worked*
Using the app to help each other in case of emergency	Alerts	*Develop a system of automatic alerts to be sent to emergency contacts; Check with network if they have heard from someone*
Reinforcement of each other’s accomplishments through the app	Use accomplishments to motivate others	*Show accomplishments e.g., with improved glucose values to encourage others*
